# Screening Various Bacterial-Produced Double-Stranded RNAs for Managing Asian Soybean Rust Disease Caused by *Phakopsora pachyrhizi*

**DOI:** 10.3390/plants15020294

**Published:** 2026-01-19

**Authors:** Yenjit R. Thibodeaux, Sunira Marahatta, Dongfang Hu, Maria Izabel Costa de Novaes, Isabel Hau, Tong Wang, Zhi-Yuan Chen

**Affiliations:** 1 Department of Plant Pathology and Crop Physiology, Louisiana State University Agricultural Center, Baton Rouge, LA 70803, USA; yraruang76@gmail.com (Y.R.T.); smarahatta@agcenter.lsu.edu (S.M.); dfhu2019@gmail.com (D.H.); izabelcnovaes@gmail.com (M.I.C.d.N.); ihau1@lsu.edu (I.H.); wtwtp@126.com (T.W.); 2 Southern Regional Research Center, Agricultural Research Service, United States Department of Agriculture (USDA/ARS), New Orleans, LA 70124, USA; 3 Shandong Peanut Research Institute, Qingdao 266100, China

**Keywords:** dsRNA, spray induced gene silencing, soybean, fungal disease management, resistance to rust disease

## Abstract

Asian soybean rust (ASR), caused by *Phakopsora pachyrhizi* (Syd.), poses a serious threat to global soybean production. The main approach to managing this disease has been through repeated fungicide applications which have reduced efficacy due to fungicide resistance. Recently, spray-induced gene silencing (SIGS) through exogenous application of double-stranded RNA (dsRNA) has emerged as a promising approach for plant disease management. In the present study, twelve different dsRNAs targeting genes important for *P. pachyrhizi* urediniospore germination, infection of the host plant or resistant to commonly used fungicides were produced in *Escherichia coli* on a large scale. Nine of these dsRNAs significantly reduced ASR severity (by 24.0% to 81.1%) and fungal biomass (50.5% to 83.1%) compared to the control when applied as a foliar spray in our growth chamber studies. Three of the most effective dsRNAs targeting an acyltransferase (ACE), cytochrome B (CYTB1) and a reductase (S12) also significantly reduced disease severity (78.2 to 82.3%) and fungal growth (79.8 to 85.4%) compared to the control in the greenhouse studies. Further investigation of the *P. pachryrhizi* urediniospore germination and hyphal growth in the presence of these dsRNAs in vitro revealed these dsRNAs reduced the spore germination rate from 72.1% to 0.0–26.6% at 4.5 h and hyphal growth from 254.0 µm to 2.7–40.5 µm at 9 h, with dsRNA targeting the *S12* gene being the most effective. These results highlight the potential of SIGS using selected dsRNAs as a sustainable strategy for managing ASR through suppressing urediniospore germination and hyphal growth.

## 1. Introduction

Soybean (*Glycine max* L.) is one of the most important agricultural crops, cultivated worldwide in about 146.8 million ha of land in 2024/2025 according to the latest report released by USDA-FAS (https://apps.fas.usda.gov/psdonline/circulars/production.pdf (accessed on 15 August 2025)). The total global soybean production in 2025 was projected to be 424.2 million metric tons (MMTs). The US soybean production is projected to reach 118.8 MMTs in 2025 from 34.8 million ha of land, which accounted for about 23.7% of the total acreage and 28.0% of worldwide soybean production.

However, global soybean production is under constant threats of various diseases, such as Asian soybean rust (ASR), caused by *Phakopsora pachyrhizi* Syd. ASR has caused substantial yield losses in Asia (10–100%) [1], and up to 90% in South American countries, such as Brazil, if left untreated [2,3]. ASR contributed to a yield loss of 6.6% in South Brazil, Paraguay, Uruguay and Argentina based on a recent estimate [4]. ASR was first reported in Japan in 1902 [5]. Later, it was detected in other Asian countries, such as China, followed by countries in Africa and South America, such as in Paraguay, in 2001 [2]. It reached North America in Louisiana for the first time in late 2004 [6].

Although soybean Plant Introduction (PI) lines with resistance to *P. pachyrhizi* have been identified through extensive screening efforts in the past several decades, such as PI200492, PI230970, PI417089A, PI576104B and PI605823 [7,8,9] and seven major resistance (*R*) genes have been identified (*Rpp1* to *Rpp7*) [9,10,11], most of these resistance genes have been overcome by the existing pathogen populations [7,12], with the exception of *Rpp6907* [13,14]. Currently, ASR is managed through repeated fungicide applications, as many as 6–7 applications per season in Brazil and other South American countries [15]. This practice has caused the development of fungicide resistance in the pathogen populations [16,17,18] significantly increasing the production cost and reducing the sustainability of soybean production in the long run [19].

Recently, successful disease suppression through the application of in vitro synthesized dsRNA (double-stranded ribonucleic acid) by Koch et al. [20] opened an RNA interference (RNAi) or gene silencing-based non-GMO approach to manage plant diseases. This foliar application of dsRNA to achieve disease suppression is called spray-induced gene silencing (SIGS). Gene silencing is a naturally occurring and evolutionarily conserved regulatory mechanism that can lead to the sequence-specific degradation of mRNA and post-transcriptional gene silencing by dsRNA to regulate plant growth and development, and to defend against viral or pathogen infections [21,22]. In eukaryotic cells, this is achieved through a series of steps involving Dicer-like proteins (DCLs), RNA-induced silencing complex (RISC), argonaute proteins and the production of small interfering RNAs (siRNA) of 21–25 nucleotides in length [23,24,25]. RNA-dependent RNA polymerase (RdRp) can further amplify the silencing effect by converting single-stranded RNAs (ssRNAs) into dsRNAs and these newly formed dsRNAs are then processed once more by Dicer-like proteins to initiate a fresh cycle of RNA silencing [25]. The SIGS uses the non-toxic, biodegradable and highly specific dsRNA to target pathogen species, making it an environmental friendly and sustainable alternative to conventional pesticide-based approaches.

Since the initial report of successful reduction in Fusarium disease on the barley leaves through dsRNA targeting fungal *CYP* genes in 2016 by Koch et al. [20], many studies have demonstrated the potential of SIGS in managing plant fungal diseases, such as how targeting the *DCL* genes reduced gray mold pathogen *Botrytis cinerea* infection on fruits and vegetables [26], and targeting the fungal-specific lanosterol 14α-demethylase gene (*CYP51*), a cytochrome P450 monooxygenase gene (*CYP*) superfamily that is essential for the biosynthesis of ergosterol crucial for membrane integrity, reduced white stem rot symptoms in canola by *Sclerotinia sclerotiorum* [27]. Similarly, dsRNAs targeting sorbitol dehydrogenase, translation elongation factor 1-α and heat shock protein 90 reduced the virulence of *Phytophthora infestans* on potato plants [28], targeting fungal argonaute 2 reduced fungal virulence in *Sclerotinia sclerotiorum* on tobacco leaves [29] and targeting carbohydrate esterase 5 in *Venturia inaequalis* reduced apple scab disease on culture plates [30]. Recently published studies from our laboratory also demonstrated that foliar application of dsRNAs targeting an acyltransferase gene (*ACE*) from *P. pachyrhizi* and that of the *Avr4* effector gene from *Cercospora* cf. *flagellaris* significantly reduced fungal growth and disease symptoms of ASR and Cercospora leaf blight on soybean, respectively [31,32,33].

**Figure 1:**
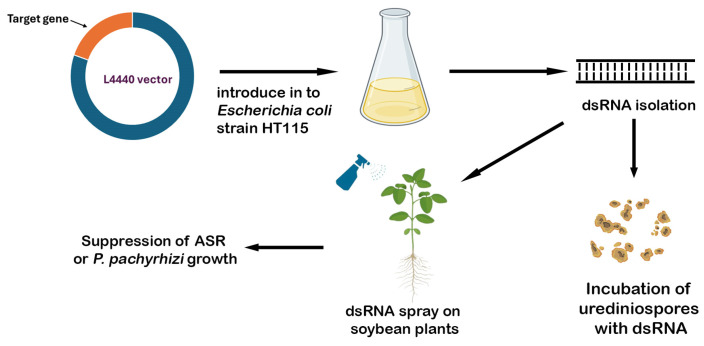
Schematic design of the overall study.

In the present study, we screened 11 additional new dsRNAs targeting various genes from *P. pachyrhizi* that were highly expressed during urediniospore germination and appressorium formation according to the previous study [34], such as different regions (*CYP3* and *CYP4*) of the eburicol 14 alpha-demethylase gene (*CYP51*); *S5*, an unknown gene encoding a putative ubiquitin protein ligase; different regions (*NH5* and *S9*) of an unknown gene encoding a hypothetical protein, different regions (*S6*, *S12* and *NH8*) of a gene encoding a putative 3-hydroxy-3 methylglutaryl-coenzyme A reductase; *S10*, a hypothetical chitin synthase gene, and different regions (*CYTB1* and *CYTB2*) of the *P. pachyrhizi* mitochondrial cytochrome B gene that is involved in mitochondrial respiration and a target of frequently used quinone outside inhibitor (QoI) fungicides. The selection of these gene targets, as well as different regions of the same target, was based on their time of expression during infection, and on the siDirect 2.0 prediction tool [35], respectively. The dsRNAs produced in a bacterial system were applied to soybean plants grown in growth chambers and a greenhouse, and to *P. pachyrhizi* urediniospores in vitro, to examine their effectiveness in reducing ASR symptoms and fungal growth both in planta and in culture (Figure 1). The objectives of the present study are to screen additional dsRNAs for their ability to suppress ASR disease severity to identify the most potent ones, and to understand the mechanism of the disease suppression through the in vitro culture assay.

## 2. Results

### 2.1. Target Gene Cloning and dsRNA Production

**Figure 2:**
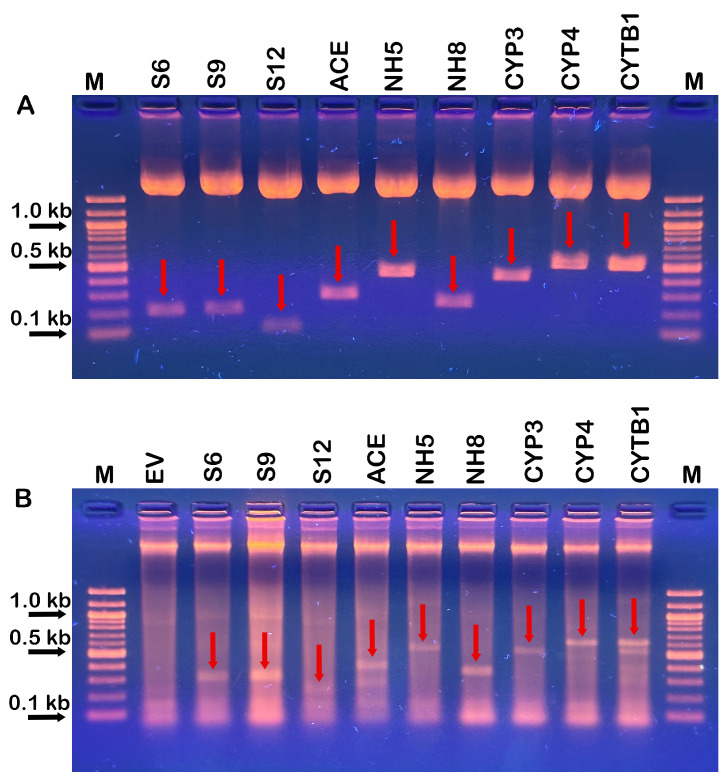
Agarose gel electrophoresis of double-digested recombinant dsRNA production vectors containing various cloned target gene fragments from *P. pachyrhizi* (**A**) and total ribonucleic acids (TNAs) isolated from *E. coli* HT115 strain containing different dsRNA overexpressing constructs (**B**). (**A**) Recombinant L4440 plasmids with different target gene fragments inserted were digested with SacI/XhoI, separated on 1.2% (*w*/*v*) agarose gel and visualized under UV after staining with GelRed. Lane M: 100 bp DNA marker; Lanes 1 to 9 are the recombinant plasmids containing the following target DNA fragments: *S6*; *S9*; *S12*; *ACE*; *NH5*; *NH8*; *CYP3*; *CYP4*; and *CYTB1*, respectively. Horizontal black arrows indicate 0.1, 0.5 and 1 kb bands. Vertical red arrows indicate the expected bands in the samples. (**B**) Total RNA (TNA) isolated from *E. coli* cells containing the dsRNA expressing vectors after IPTG induction to visualize the production of gene-specific dsRNAs. Lane M: 100 bp DNA marker; Lanes 1 to 10 contain TNA isolated from bacterial cells containing empty L4440 vector (EV); or L4440 vector with one of the following target gene fragments: *S6*; *S9*; *S12*; *ACE*; *NH5*; *NH8*; *CYP3*; *CYP4*; and *CYTB1*. Horizontal black arrows indicate 0.1, 0.5 and 1 kb bands. Vertical red arrows indicate the expected dsRNA bands in the isolated RNA samples.

The target DNA fragments needed for the dsRNA production in *E. coli* were PCR amplified using gene-specific primers (Supplement Table S1), digested and cloned into the dsRNA production vector L4440, as described in Material and Methods. In order to confirm the successful target gene insertion, recombinant plasmid DNAs were isolated from colonies identified to be positive via colony PCR and double digested with SacI/XhoI and the expected sizes of the target DNA inserts in the range of 0.25 to about 0.8 kb were observed in the agarose gel after electrophoresis (Figure 2A). The positive cloning of the correct gene targets was also verified through plasmid sequencing.

The total RNA or total ribonucleic acid (TNA), isolated from bacterial cultures after inducing the gene-specific dsRNA production through the addition of IPTG, were visualized using agarose gel electrophoresis, which confirmed the sucessful production and extraction of the gene-specific dsRNAs (Figure 2B). The dsRNA size appeared slightly larger than the expected size due to the transcription of an additional 30–40 bp of the sequences in the L4440 vector from the T7 promoters to the corresponding restriction enzyme sites. The yield of TNA ranged from 3.85 to 6.60 mg per g of fresh weight of harvested bacterial cells. Approximately 10–12% of the TNA is estimated to be the specific dsRNA based on the BioRad Gel densitometer and analysis software (Figure 2B).

### 2.2. Evaluate the Efficacy of Various dsRNAs in Suppressing Asian Soybean Rust in Soybean Under Growth Chamber Conditions

**Figure 3:**
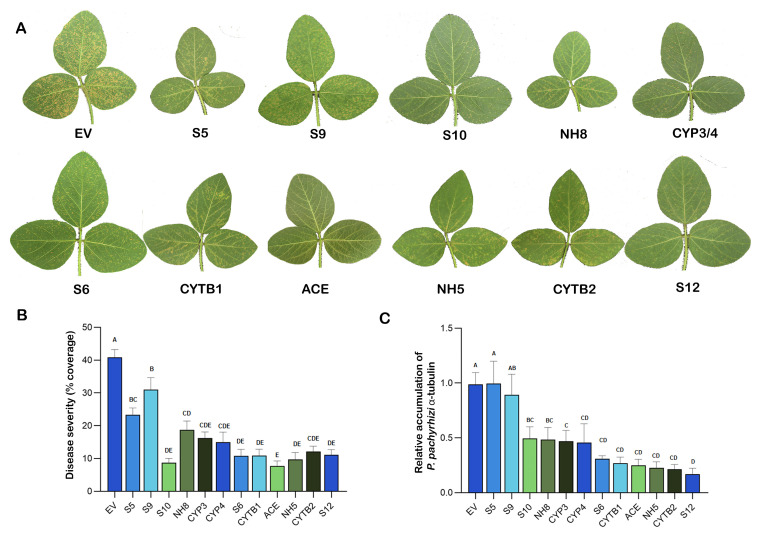
Growth chamber evaluation of the effect of different dsRNAs in suppressing soybean rust disease. (**A**) (Visual soybean rust disease symptoms of representative leaves that had been treated with various dsRNAs targeting *P. pachyrhizi* genes with dsRNA isolated from empty vector (EV) as a negative control. The visual symptoms of CYP3 and CYP4 dsRNA-treated leaves were similar, and therefore, only one image labeled CYP3/4 was included in the figure. Photos were taken 14 days after total RNA treatments and inoculation with urediniospores of *P. pachyrhizi* (1 × 10^4^ per mL). (**B**) Rust disease severity of soybean leaves was quantified using ImageJ (Fiji). (**C**) Relative accumulation of *P. pachyrhizi* α-tubulin gene (as an indicator of fungal growth or biomass) was quantified by real-time PCR and normalized to soybean ubiquitin gene (as an indicator of soybean biomass), with the EV control treated leaves set to 1. Data are presented as means ± standard error of the mean (SEM). One-way ANOVA followed by Tukey’s multiple comparison test was used to separate the differences. Bars with different letters are significantly different at *p* ≤ 0.05.

To evaluate the efficacy of dsRNA for the disease suppression on soybean plants, 11 different new dsRNAs and ACE and empty vector (EV) dsRNAs from a previous study were sprayed onto soybean plants grown in the growth chamber two hours prior to inoculation with *P. pachyrhizi* urediniospores. Twenty-four hours post-inoculation, the dsRNAs were sprayed on soybean plants again. Disease severity and fungal biomass accumulation were quantified at 14 days after the inoculation (Figure 3). On the 14th day, clear differences in disease development were observed among different dsRNA treatments. Trifoliate leaves treated with EV dsRNA had the highest pustule density (Figure 3A). In contrast, trifoliate leaves treated with gene-targeting dsRNAs showed visibly less pustule compared to the EV control (Figure 3A). Quantitative analysis of disease severity using ImageJ (Fiji), version 1.52p software revealed a 24.0–81.2% reduction in disease severity as compared to the EV control (Figure 3B). The highest reduction in disease severity was 81.2%, exhibited by ACE dsRNA, followed by S10 (78.6%), NH5 (76.3%), S6 (73.6%), CYTB1 (73.3%), S12 (72.8%), CYTB2 (70.2%), CYP4 (63.3%), CYP3 (60.1%), NH8 (54.1%), S5 (42.8%) and S9 (24.0%). Moreover, the trifoliate leaves treated with EV dsRNA displayed the highest fungal DNA accumulation based on qPCR, consistent with visual assessment and disease severity (Figure 3C). A 50.5 to 83.1% reduction in fungal biomass was observed in the trifoliate leaves treated with gene-targeting dsRNAs compared to the EV control, except for S5 and S9 dsRNAs, which did not show a significant reduction in fungal biomass. The trifoliate leaves treated with S12 dsRNA showed the highest reduction of 83.1% in fungal biomass compared to the EV control. Similarly, CYTB2 reduced the fungal biomass by 78.6%, NH5 by 77.4%, ACE by 75.2%, CYTB1 by 73.2%, S6 by 69.2%, CYP4 by 54.2%, CYP3 by 53.1%, NH8 by 51.4% and S10 by 50.6%. These results confirmed that the disease suppression by the different dsRNAs was due to the inhibition of the growth of the pathogen, rather than masking disease symptoms by other factors.

### 2.3. Further Evaluation of the Three Most Effective dsRNAs in Suppressing Asian Soybean Rust in Soybean Under Greenhouse Conditions

**Figure 4:**
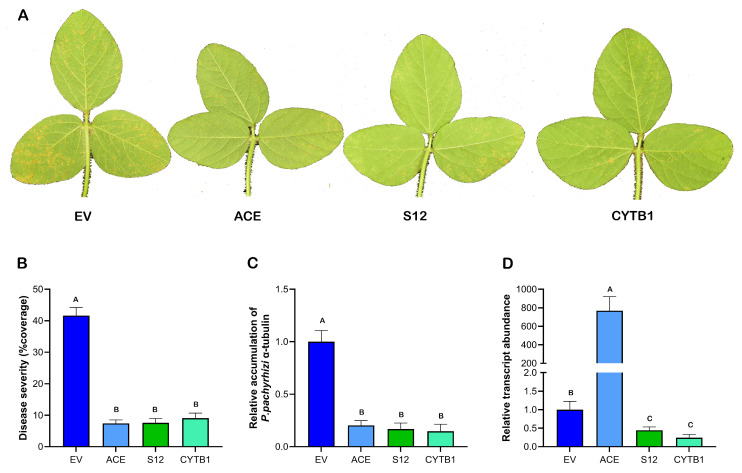
Greenhouse evaluation of different dsRNAs in suppressing Asian soybean rust disease. (**A**) Visual symptoms of soybean rust disease on representative leaves that had been treated with dsRNAs targeting ACE, S12 and CYTB1. The plants leaves treated with dsRNA from empty vector (EV) were used as a negative control. Photos were taken 14 days after total RNA treatments and inoculation with urediniospores of *P. pachyrhizi* (1 × 10^4^ per mL). (**B**) Asian soybean rust disease severity of soybean leaves was quantified using ImageJ (Fiji). (**C**) Relative accumulation of *P. pachyrhizi* α-tubulin gene (as an indicator of fungal growth or biomass) normalized to soybean ubiquitin gene (as an indicator of soybean biomass) was quantified by real-time PCR with the EV set at 1. (**D**) Relative transcript levels of target gene in soybean leaves 2 weeks post-inoculation with *P. pachyrhizi*. Values are expressed relative to the endogenous *P. pachyrhizi* α-tubulin gene with the EV set at 1. Data are presented as means ± standard error of the mean (SEM). One-way ANOVA followed by Tukey’s multiple comparison test was used to separate the differences. Bars with different letters are significantly different at *p* ≤ 0.05.

The top three most effective dsRNAs (ACE, S12 and CYTB1) in suppressing ASR identified via the above well-controlled growth chamber study were further validated with soybean plants inoculated with *P. pachyrhizi* urediniospores under greenhouse conditions to better mimic the natural field microclimate. Disease severity and fungal biomass accumulation were quantified at 14 days after the inoculation (Figure 4). Like the growth chamber study, on the 14th day, clear differences in disease development were observed among the different dsRNA treatments. Trifoliate leaves treated with EV dsRNA had the greatest number of pustules per leaflet based on visual assessment when compared to the leaves treated with gene-targeting dsRNAs (Figure 4A). Quantitative analysis of disease severity revealed a significant reduction in disease severity when compared to the control leaves treated with EV dsRNA (Figure 4B). The highest reduction was 82.3%, exhibited by ACE dsRNA, followed by S12 (81.8%) and CYTB1 (78.2%). Moreover, the trifoliate leaves treated with gene-specific dsRNAs had a 79.8% to 85.4% reduction in fungal biomass in comparison to the control in our qPCR analysis, with CYTB1 dsRNA showing the highest suppression in fungal biomass, followed by S12 (83.4%) and ACE (79.8%). This suppression in ASR disease severity and fungal biomass by our dsRNA treatments is also associated with a significant reduction of 54.5 to 76.0% in target transcript abundance in the leaves treated with S12 or CYTB1 dsRNA, respectively, in comparison to the control leaves treated with EV dsRNA. The expression of the ACE target gene increased over 852-fold when compared to that of the control in our repeated studies, which is quite puzzling. This might be the result of genetic compensation or transcriptional adaptation, which will be explained further in the discussion section. These results show the potential of the foliar application of these dsRNAs for the practical management of Asian soybean rust disease under the field settings.

### 2.4. In-Culture Assays to Evaluate the Effectiveness of the Most Effective dsRNAs in Suppressing Spore Germination and Fungal Growth

**Figure 5:**
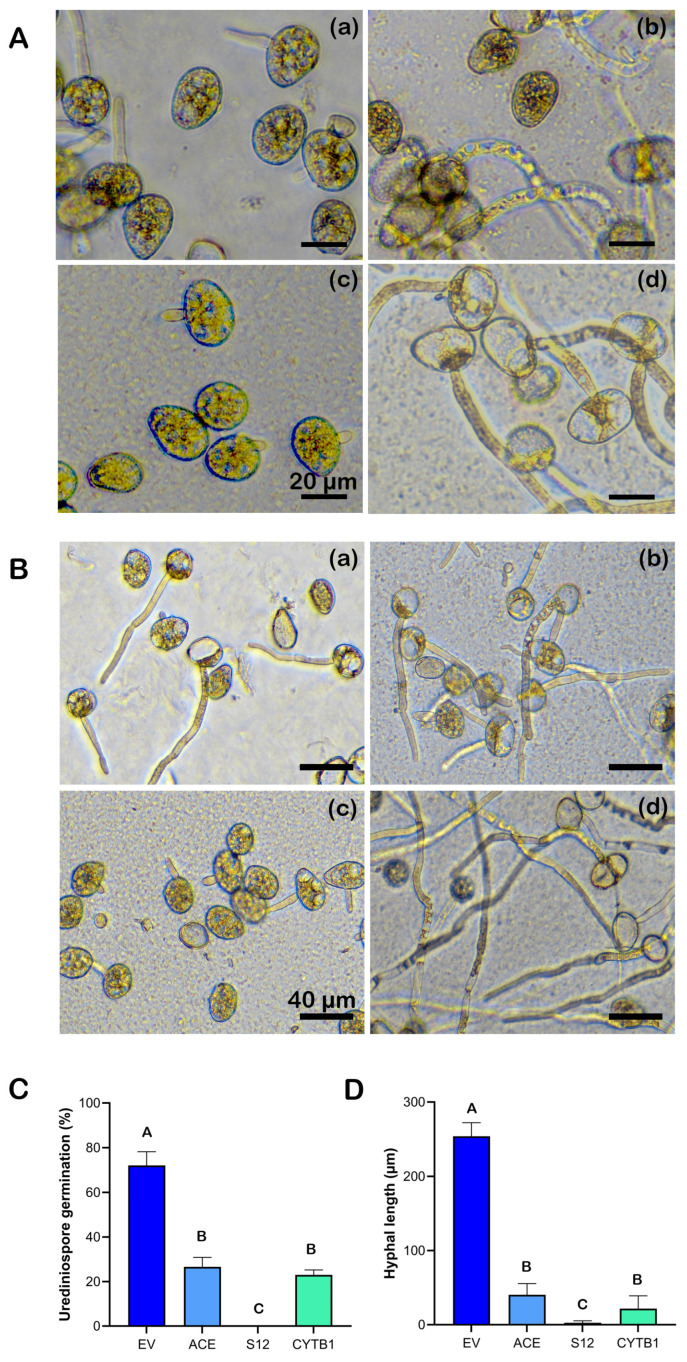
The visual (**A**,**B**) and quantitative (**C**,**D**) effects of different dsRNA treatments on spore germination (**A**,**C**) at 4.5 h and hyphal length (**B**,**D**) at 9 h of *P. pachyrhizi* after in vitro incubation with various dsRNAs. *P. pachyrhizi* urediniospores were germinated in H_2_O with different dsRNAs targeting ACE (**a**), CYTB1 (**b**), S12 (**c**) and EV (empty vector control) (**d**). The final concentration of total RNA in water was 200 ng/μL. Over 100 of germinated or ungerminated spores were counted and over 50 hyphae were measured from the germinated spores to quantify the effects of dsRNA on spore germination (**C**) and hyphal growth (**D**). Scale bar represents 20 μm (**A**) and 40 μm (**B**). The bar represents mean values ± standard error of the mean of one representative experiment. One-way ANOVA followed by Tukey’s multiple comparison test was performed. Bars with different letters are significantly different determined at *p* ≤ 0.05.

To understand the mechanism of the dsRNA in suppressing the pathogen growth and disease development on soybean leaves, *P. pachyrhizi* urediniospores were incubated in vitro with the most effective gene-targeting dsRNAs (ACE, S12 and CYTB1) with EV dsRNA as a control to determine their effects on spore germination and hyphal growth. A clear variation in the effectiveness among the gene-targeting dsRNAs in suppressing spore germination at 4.5 h or hyphal growth at 9 h was observed when examined under a microscope, with S12 being the most effective one (Figure 5). At 4.5 h after incubation, the control urediniospores that were treated with dsRNA extracted from bacterial culture containing only the empty vector (EV dsRNA) had a germination rate of 72.1 ± 6.2%. The germination rate of urediniospores treated with gene-targeting dsRNAs was significantly lower, ranging from 26.6 ± 4.24% for ACE dsRNA-treated spores, followed by CYTB1 (23.0 ± 2.3%) to no germination observed in spores treated with S12 dsRNA (Figure 5A,C). Other dsRNAs were also screened for their potential effects in suppressing *P. pachyrhizi* urediniospore germination and hyphal growth (Figure S1). CYP4 dsRNA reduced the spore germination to 23.9 ± 3.3% and NH5 to 22.0 ± 4.1%, as compared to 72.1 ± 6.2% of the urediniospore germination rate in the EV dsRNA-treated control (Figure S1). Nine hours after incubation, the effect of different dsRNA treatments on the hyphal growth of *P. pachyrhizi* was assessed. The fastest hyphal growth was observed from urediniospores treated with EV dsRNA. The hyphal length was measured at 254.0 ± 18.4 μm, while the hyphal growth from urediniospores treated with gene-targeting dsRNAs was significantly reduced. The average hyphal length was 40.5 ± 15.2 μm for those spores treated with ACE dsRNA, to 21.7 ± 17.4 μm for spores treated with CYTB1 dsRNA. The slowest hyphal growth was observed from urediniospores treated with S12 dsRNA, which was measured at 2.7 ± 2.7 μm on average (Figure 5B,D). The hyphal length from urediniospores treated with other dsRNAs was also significantly shorter (62.1 ± 13.3 μm for CYP4-treated spores and 15.9 ± 4.0 μm for NH5-treated spores) in comparison to the control treated with EV dsRNA (Figure S1). These results indicate the reduced ASR disease severity and fungal growth in dsRNA-treated soybean leaves is likely due to the inhibitory effects of the different dsRNAs on the germination and hyphal growth of *P. pachyrhizi* urediniospores.

## 3. Discussion

A crucial factor for the success of RNA interference-based crop protection is the accurate identification of the appropriate gene targets to silence. The fungal pathogenicity-related genes are frequently selected as targets for effective disease management through SIGS [36,37]. Pathogens often overexpress different catalases to counteract the reactive oxygen species (ROS) produced by host plants during infection [38,39]. Hence, these catalases can also be potential targets. Knocking these down can reduce the virulence of the pathogens [36]. Also, genes targeted by commonly used fungicides have been successfully used in fungal disease suppression via SIGS, such as the *CYP51*, which encodes cytochrome P450 lanosterol C-14α-demethylase and is a key target of triazole-based fungicides such as DMIs to inhibit ergosterol biosynthesis and thereby disrupting fungal membrane integrity [20,37]. Another potential target is *CYTB*, which encodes a mitochondrial protein cytochrome b involved in respiration and energy production and is frequently targeted by QoI fungicides to starve the fungus and make it die rapidly [20,37]. An added benefit of targeting these genes via SIGS is that it can reduce the pathogen populations that have developed resistance to DMI or QoI fungicides [40], which can greatly improve the efficacy of these fungicides.

In our previous study, we reported the identification of dsRNAs targeting *ACE* and a couple of other genes that were very effective in suppressing ASR when sprayed onto detached soybean leaves [31]. However, combining dsRNAs targeting different genes may be needed to maintain the effectiveness of dsRNA treatment and to minimize the chance of pathogens developing resistance to the existing dsRNAs, although such an incidence has not been observed in fungal pathogens [41]. It has been reported in Colorado potato beetles (*Leptinotarsa decemlineata* Say) after repeated exposure to high doses of dsRNA-coated leaves, which increased their tolerance to the target dsRNA by 11,100-fold [42]. This might be particularly important for effective management of ASR through SIGS due to the nature of *P. pachyrhizi*, which introduces high rate of random mutations during vegetative reproduction and develops resistance quickly to commonly used fungicides [43].

In the present study, different regions of the cytochrome b gene (*CYTB1* and *CYTB2*) and cytochrome P450 lanosterol C-14α-demethylase gene (*CYP3* and *CYP4*) were selected as potential targets for suppression through dsRNAs to determine whether they can reduce ASR disease severity in our growth chamber studies. All of these dsRNAs were equally effective in significantly reducing the disease severity and fungal biomass compared to the EV control (Figure 3B,C). CYTB1 dsRNA was further screened in the greenhouse for its potential suppression in field-like settings, and similar results were reproduced (Figure 4).

Several other targets were also selected for suppression via SIGS in this study, such as genes that were upregulated during the spore germination and appressoria formation of *P. pachyrhizi* [34]. Disrupting the expression of these genes via host-induced gene silencing (HIGS) prevented or slowed haustoria formation and disease establishment in other pathogens, like *Puccinia striiformis* f. sp. *tritici* [44], *Blumeria graminis* f. sp. *hordei* [45] and *Bremia lactucae* [46]. In our SIGS study, targeting these early expressing genes also led to reduced disease severity (Figure 3). One of the most effective dsRNAs identified in this study is S12, which targets the expression of a putative 3-hydroxy-3-methylglutaryl-coenzyme A reductase. It suppressed the fungal biomass accumulation by at least 81% in both growth chamber and greenhouse studies when compared to the EV dsRNA-treated controls (Figure 3B and Figure 4C).

In order to determine whether this reduced disease severity and fungal growth in the dsRNA-treated and inoculated soybean leaves in the greenhouse study was the result of gene silencing caused by gene-specific dsRNAs, the target gene expression in the treated soybean leaves was determined through qPCR with primers outside the dsRNA region. The expression of *S12* and *CYTB1* genes was reduced significantly, as expected. However, the expression of *ACE* following dsRNA treatment increased over 852-fold. To rule out any possible mistakes, a second set of real-time PCR primers was used, and similar result of overexpression was observed (Figure S2). A similar magnitude of compensatory transcriptional upregulation has been reported in several other studies [47,48,49]. This might be a phenomenon of “transcriptional adaptation” triggered by target mRNA degradation [50]. However, other factors, such as stress-induced overexpression, cannot be ruled out [51], due to the critical role of *ACE* in membrane lipid metabolism.

To gain mechanistic insights of the disease suppression, spore germination and hyphal length were evaluated at 4.5 and 9 h, respectively, after the incubation of *P. pachyrhizi* urediniospores in the presence of dsRNA. Compared to the 72% spore germination rate in the EV dsRNA-treated control, CYTB1 dsRNA treatment significantly reduced the spore germination rate to 23.0% (Figure 5A,C). Moreover, the hyphal growth from the spores treated with CYTB1 dsRNA was reduced by 91.5% after 9 h of incubation when compared to the EV dsRNA-treated control (Figure 5B,D), indicating that CYTB1 dsRNA effectively reduced ASR disease symptoms and fungal infection in soybeans through reducing the spore germination and limiting the growth of fungal hyphae during the initial stage of infection to limit the pathogen’s ability to establish in the soybean leaves. S12 is the most effective dsRNA in suppressing *P. pachyrhizi* urediniospore germination and hyphal growth (over 98%) in vitro in comparison to the EV dsRNA-treated control (Figure 5). It showed the same (based on in planta assays) or better (based on the in vitro assay) effectiveness when compared to the previously reported ACE dsRNA [31], which suppressed the fungal growth in vitro by 84% in comparison to the EV dsRNA-treated control. Therefore, the dsRNAs which exhibited similar if not better effectiveness in suppressing ASR than the previous reported ACE have been identified in this study.

In order to identify the most effective dsRNA and to avoid off-target effects, the selected target genes were analyzed via siDirect 2.0 and multiple regions for some of the target genes were selected for dsRNA production. Several previous studies reported that dsRNAs targeting different regions of the same gene, either overlapping or non-overlapping, had different silencing efficiencies, possibly due to variation in siRNA production from different regions of the target genes [29,52,53]. Similar variations in efficacy were also observed in our study. For example, S9 and NH5 are parts of the same gene; however, S9 was not as effective as NH5 in suppressing ASR in the growth chamber study. Similarly, S12 dsRNA was more effective than both NH8 and S6 in the growth chamber study.

Non-target dsRNA, such as those targeting the *GFP* gene, that is often used as a control in SIGS experiments [20,54,55], has been reported to cause PAMP (pathogen-associated molecular pattern)-triggered immunity (PTI) and altered the expression of many host genes [56,57]. For this reason, the dsRNA targeting *GFP* was not included in this study, after an initial assessment that observed a suppression on ASR disease symptoms at a concentration of about 250 µg/mL TNA in comparison to leaves treated with EV dsRNA. This concentration is similar to the 20 µg/mL used by Koch et al. [20], considering only 10–12% of our TNA is gene-specific dsRNA. It is also similar to the 300 μg/mL of total bacterial RNA expressing hpRNA, used to achieve successful suppression of Sclerotinia stem rot in canola by Azizi and del Río Mendoza [58]. However, Zheng et al. [59] observed a sequence-unspecific effect of dsRNA at 10 µg/mL on *Magnaporthe oryzae*. We confirmed that the target gene expression between the non-treated leaves and the EV dsRNA-treated leaves was not significantly different through real-time PCR, demonstrating the suppression was not due to stress responses and ROS production of *P. pachyrhizi* caused by the presence of sequence-independent bacterial RNAs.

In SIGS, how exactly the applied dsRNA is taken up by plants or pathogens and converted to siRNA is still unclear. The exogenously applied dsRNAs have at least two potential pathways to reach fungal cells from plant surfaces: one is through plant cells, and the other is entering the fungal cells directly. For the dsRNAs entering the plant cells, some are cleaved into siRNAs in the cytoplasm by plant Dicer-like proteins (DCLs), and these siRNAs are then secreted from the plant cell in exosomes or extracellular vesicles, which can be taken up by the invading pathogen to initiate gene silencing [21]. Fungal haustoria or similar structures are likely the main sites for siRNA uptake from host plant cells, as siRNA-containing vesicles accumulate around these structures [60]. Simultaneously, some of these topically applied dsRNAs can be directly taken up by fungal cells during the infection of plant tissues via clathrin-mediated endocytosis, such as in *Sclerotinia sclerotiorum* [60]. Once inside fungal cells, the dsRNAs are processed into siRNAs by the respective DCLs [61,62]. The sense strand of the siRNA duplex is degraded, while the antisense (guide) strand interacts with RISC and attaches to the target mRNA sequence via base pairing, initiating the degradation of the targeted mRNA and thus the key function of the targeted pathogen [63].

In summary, this study has identified additional targets for effective management of ASR via SIGS and confirmed the suppression was gene-specific and was through reducing *P. pachyrhizi* urediniospore germination and hyphal growth to limit the pathogen establishment and growth on dsRNA-treated soybean leaves. These additional gene targets can ensure the sustainability and durability of SIGS in managing ASR. However, poor uptakes of dsRNAs by leaf tissue and low stability of dsRNA under natural environmental conditions remain the main challenges for practical and large-scale plant fungal disease management via SIGS. Recent studies have demonstrated that nanomaterials, such as layered double hydroxides and carbon quantum dots, as well as adjuvants, can improve the uptake and stability of applied dsRNA under greenhouse conditions [63,64,65], which are what we will be exploring in the future research to make this dsRNA-based disease management approach more practical. Future research will also explore the feasibility of one dsRNA spray of these three most effective dsRNAs as a preventive or curative treatment in managing ASR.

## 4. Materials and Methods

### 4.1. Selecting the Target Genes

For selecting potential target genes to suppress through dsRNA for managing Asian soybean rust, the published transcriptomic data were searched [31,34,66], and the following genes were selected based on their high expression during spore germination or infection of host and their potential involvement in haustoria or appressoria formation, such as an acyltransferase gene (*ACE*), a putative reductase, *NH5* and *NH8* [34,66]. Also, the genes targeted by commonly used quinone outside inhibitor (QoI) and demethylation inhibitor (DMI) fungicides, such as the cytochrome B genes (*CYTB1* and *CYTB2*) involved in mitochondrial respiration and the eburicol 14 alpha-demethylase genes (*CYP3* and *CYP4*) involved in maintaining the fungal membrane integrity, were selected [15,67,68]. Please refer to Table 1 for details on the selected gene targets and their potential functions based on the literature.

### 4.2. Clone the Target Genes from Phakopsora pachyrhizi into a dsRNA Expression Vector

Fragments of the target genes, *ACE, S5, S6, S9, S10, S12, NH5, NH8, CYTB1, CYTB2, CYP3* and *CYP4* were PCR amplified using the primers listed in Table S1 with 1 µL of 50 ng/µL of cDNA as a template, according to [69]. The expected DNA bands were excised from the 1% agarose gel after electrophoresis and purified using the QIAquick Gel Extraction Kit (Qiagen, Germantown, MD, USA) according to the manufacturer’s protocol. The resulting PCR products and the L4440 vector were digested with the same SacI/XhoI restriction enzymes (New England Biolabs, Ipswich, MA, USA) and purified using the QIAquick PCR Purification Kit (Qiagen). Ligation was performed using T4 DNA ligase (New England Biolabs, Ipswich, MA, USA) at 25 °C for 2 h following the manufacturer’s protocol. The ligated products were transformed into *Escherichia coli* HT115 (DE3) competent cells and plated on LB agar plates supplemented with 100 µg/mL ampicillin and 12.5 µg/mL tetracycline (Sigma-Aldrich, St. Louis, MO, USA). Ten colonies per construct were selected from these plates after overnight incubation at 37 °C and screened for the presence of the insert through plasmid extraction by QIAprep Spin Miniprep Kit (Qiagen) and SacI/XhoI double digestion. Cloning of the intended target genes was confirmed through the analyzing of digested products on a 1% agarose gel and sanger sequencing using primers from the vector and correct alignment with the reference sequences using NCBI BLAST (*nt*/*nt* database) via the NCBI web-based BLAST interface.

### 4.3. dsRNA Induction and Total RNA Isolation

A protocol modified from Zivanovic and Chen [32] was used for the total RNA (TNA) extraction. Briefly, 1 L of LB broth of bacterial culture containing the L4440 vector with the target gene fragment inserted was incubated at 37 °C and 200 rpm for 3.5–4.0 h and the isopropyl β-D-1-thiogalactopyranoside (IPTG) (Sigma-Aldrich) was added to the culture to a final concentration of 0.4 mM for the dsRNA induction for an additional 5 h. The bacterial cells were harvested and resuspended in 0.8% Sodium Dodecyl Sulphate (SDS) solution to lyse through a French Press at 4 °C three times at a pressure range of 1000–1250 psi. TNA was extracted from lysed cells using the chloroform–ethanol precipitation method. The TNA concentration was determined using a NanoDrop 1000 Spectrophotometer (Thermo Fisher Scientific, Wilmington, DE, USA) and the dsRNA content in the TNA was estimated through agarose gel analysis before being stored at −80 °C until use. The use of TNA instead of purified dsRNA is to make this dsRNA-based approach more practical and economical.

### 4.4. Growth Chamber Evaluation of Different dsRNAs in Suppressing Asian Soybean Rust in Soybean

To examine the effect of dsRNA of different target genes from Table 1 against soybean rust disease, soybean cv (Syngenta S42-B9XS) were grown in a 2 L pot containing Sun Gro professional growing mix (Sun Gro Horticulture, Agawam, MA, USA) in a growth chamber that was set at the temperature 25 °C, relative humidity 90% and a 16 h light/8 h dark condition. Soybean plants at the V3–V4 growth stage were sprayed with 1.0 mL of 250 ng/µL TNA of specific dsRNA per plant on the first two trifoliate leaves. Four to five soybean pots (two plants in each pot) as individual replicates underwent specific dsRNA treatments. Approximately two hours post-application, each soybean plant received a 1.0 mL spray of *P. pachyrhizi* urediniospores (1 × 10^4^ per mL) suspended in sterile water containing 0.01% Tween 20 and was kept in dark conditions with 100% humidity for 24 h to ensure successful inoculation. The dsRNA treatment was applied one more time 24 h post-inoculation. The use of sprays before and after pathogen inoculation is to provide both preventive and curative protection of soybean plants. Evaluation of soybean rust disease development was performed 14 days post-inoculation. Visual assessment of disease severity was based on the percentage of total leaf area covered with rust pustules through ImageJ (Fiji), version 1.52p analysis of the same two trifoliate leaves from each plant [70]. Disease severity was expressed as percentage of leaf area with disease symptoms. For quantifying fungal biomass, genomic DNA was extracted from the soybean leaf using the hexadecyltrimethylammonium bromide (CTAB) method [71]. Relative accumulation of the *P. pachyrhizi* α-tubulin gene to the soybean ubiquitin gene was quantified using the QuantStudio™ 6 Flex Real-Time PCR System (Applied Biosystems, Thermo Fisher Scientific, Waltham, MA, USA) with TaqMan™ Multiplex Master Mix chemistry (Applied Biosystems). Reactions were performed in a 20 µL volume containing 1× TaqMan Master Mix, 1 µM each of forward and reverse primers, 0.5 µM TaqMan probe, and 500 ng of genomic DNA template. PCR amplification conditions consisted of an initial denaturation at 95 °C for 10 min, followed by 40 cycles of 95 °C for 15 s and 62 °C for 60 s. Relative gene accumulation was calculated using the 2^-ΔΔCt^ method [72]. The selection of the ubiquitin gene of soybeans and α-tubulin from *P. pachyrhizi* as a reference was based on previous published studies [73,74], which showed stable expression during *P. pachyrhizi* infection of soybeans. The primers used in the quantification are provided in Table S1. This growth chamber study was repeated 4–6 times for each of the target genes. The data reported were the average from all the repeats.

### 4.5. Greenhouse Evaluation of Different dsRNAs in Suppressing Asian Soybean Rust in Soybean

To validate the result from the controlled growth chamber in semi-natural conditions, the three most potent dsRNAs were examined in the greenhouse. Soybean plants S42-B9XS were grown as above. The plants were maintained under natural sunlight with supplemental lighting used when daylength <12 h, relative humidity 60–80% with automated fans and side vents and a diurnal temperature of 18–28 °C. The spray of TNA, inoculation, quantification of disease severity and fungal biomass accumulation were the same as in 4.4. For the target gene expression analysis, RNA was extracted from infected leaves using an RNeasy Plant Mini Kit (Qiagen). The expression of the target gene, normalized to the *P. pachyrhizi* α-tubulin gene, was quantified using SYBR™ Green Universal Master Mix (Applied Biosystems) in QuantStudio™ 6 Flex Real-Time PCR System (Applied Biosystems) under the same PCR conditions described above and calculated using the 2^-ΔΔCt^ method. This greenhouse experiment was repeated 3 times.

### 4.6. In-Culture Assay to Evaluate the Effectiveness of Different dsRNA in Suppressing Spore Germination for Fungal Growth

In-culture assay was performed in a 48-well microtiter plate with a total volume of 300 µL in each well by mixing 200 µL of 300 ng/µL of TNA of each gene and 100 µL of 1 × 10^5^ per mL of *P. pachyrhizi* urediniospores. The final TNA concentration in each well was 200 ng/µL. Each treatment had three replicates. The plate was incubated in room temperature for 9 h. At 4.5 and 9 h, pictures were taken via a Nikon T2i microscope for calculating spore germination and measuring hyphal growth from the germinated spores, respectively. The spores are considered germinated if the germ tube is longer than the diameter of the urediniospore, according to [75]. Over 100 germinated or ungerminated spores were counted and spore germination was calculated by dividing the number of germinated spores to the total spores, expressed as a percentage. For the hyphal length measurement, over 50 hyphae protruding from the germinated spores were measured in µm. The experiment was performed 3 times.

### 4.7. Statistical Analysis of Data

Statistical analyses were performed using one-way ANOVA followed by Tukey’s multiple comparison test in GraphPad Prism (version 10.2.3; GraphPad Software, Boston, Massachusetts, USA). Treatment effects were considered statistically significant at *p* ≤ 0.05. All results are presented as means ± standard error of the mean (SEM), unless otherwise noted.

## Figures and Tables

**Table 1:** Target genes for dsRNA production to suppress Asian soybean rust disease in soybean.

Targets	Size (bp)	Function	References
*ACE*	351	Acyltransferase or thiolase, involved in spore germination and germ tube emergence	[31]
*S5*	338	A putative ubiquitin protein ligase, involved in regulating proteins in appressorium development	[66]
*S6* *	326	A putative 3-hydroxy-3-methylglutaryl-coenzyme A reductase, involved in appressorium and early infection structure formation	[34]
*S12* *	244		
*NH8* *	536		
*S9* ^†^	312	A hypothetical conidiation-related protein (CRP_6), involved in spore germination and germ tube elongation	[34]
*NH5* ^†^	524		
*S10*	373	A hypothetical class V chitin synthase, involved in spore germination, initiation of infection structure and host–pathogen signaling	[66]
*CYTB1*	595	Cytochrome B in mitochondrion, involved in cellular respiration and ATP synthesis	[67]
*CYTB2*	331		
*CYP3*	594	Eburicol 14 alpha-demethylase for the biosynthesis of ergosterol fungal cell membrane integrity and function	[15]
*CYP4*	840		

*: *NH8*, *S6* and *S12* are part of the same gene, *S6* is more towards the 3′end and *S12* is more toward the 5′end with no overlap between the two dsRNA. However, the *NH8* sequence encloses all of the *S12*, and only has an overlapping primer sequence with *S6*; ^†^: *NH5* and *S9* are also of the same gene with 3′end portion of the *NH5* enclosing the smaller *S9* region.

## Data Availability

The original contributions presented in this study are included in the article. Further inquiries can be directed to the corresponding authors.

## Supplementary Materials

The following supporting information can be downloaded at https://www.mdpi.com/article/10.3390/plants15020294/s1, Figure S1: The visual (**A**,**B**) and quantitative (**C**,**D**) effects of additional dsRNA (NH5 and CYP4) treatments on spore germination (**A**,**C**) at 4.5 h and hyphal length (**B**,**D**) at 9 h of *Phakopsora pachyrhizi* after in vitro incubation with dsRNA; Figure S2: Relative expression of ACE amplified from another qPCR primer set. Table S1: Primers used for amplification, cloning of the target gene fragments from *P. pachyrhizi,* for quantification of fungal biomass and for quantification of target gene expression.

## Abbreviations

The following abbreviations are used in this manuscript:

DMI	Demethylation Inhibitor
EV	Empty vector
HIGS	Host-Induced Gene Silencing
QoI	Quinone Outside Inhibitor
SIGS	Spray-Induced Gene Silencing
TNA	Total Ribonucleic Acid
