# Supplementary material for: Screening Various Bacterial-Produced Double-Stranded RNAs for Managing Asian Soybean Rust Disease Caused by Phakopsora pachyrhizi
Source: Plants (Basel). 2026 Jan 19;15(2):294. doi: 10.3390/plants15020294 (PMC12845184; doi:10.3390/plants15020294)
Supplement: Supplementary file 1 [file plants-15-00294-s001.zip › plants-4070109-supplementary.pdf]

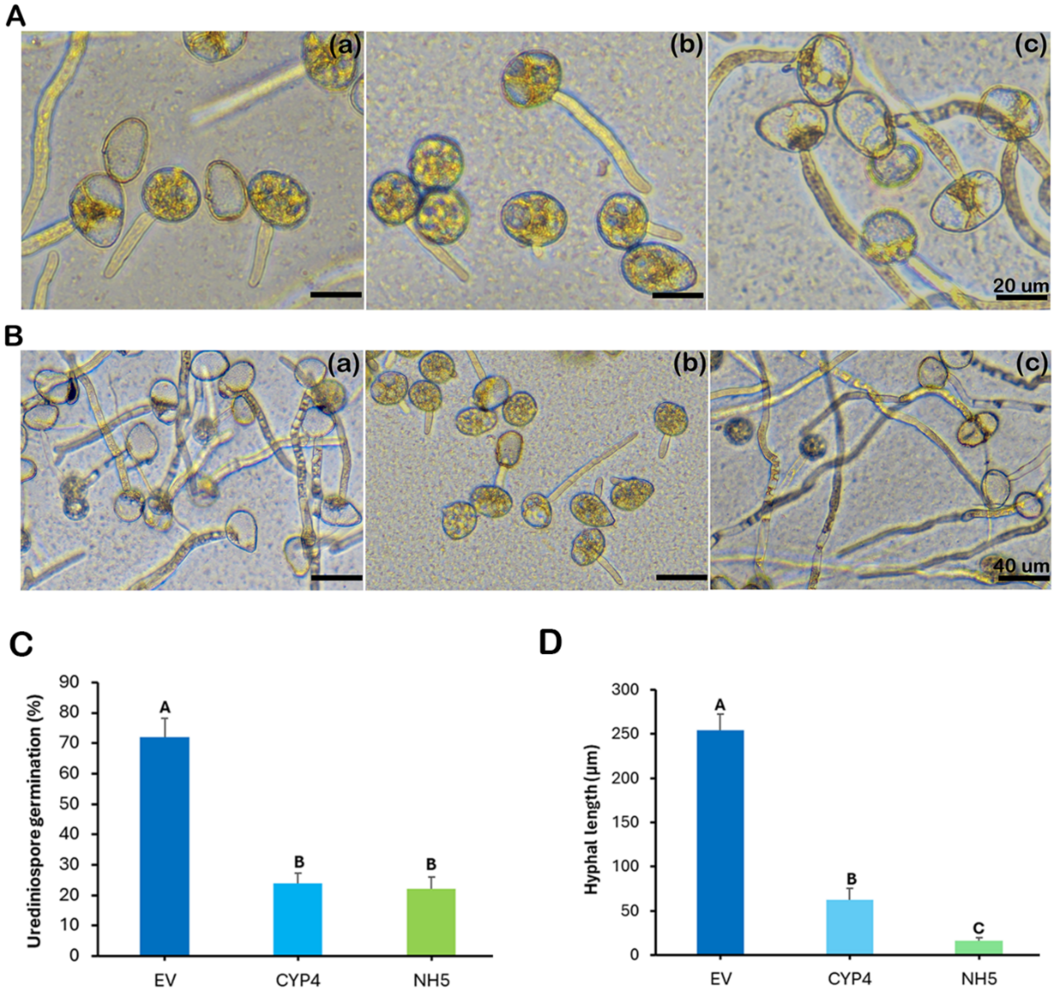

Figure S1: The visual (**A, B**) and quantitative (**C, D**) effects of additional dsRNA (NH5 and CYP4) on spore germination (A, C) at 4.5 hours and hyphal length (B, D) at 9 hours of *P. pachyrhizi* after *in vitro* incubation with dsRNA. *P. pachyrhizi* urediniospores were germinated in H_2O with different dsRNAs targeting CYP4 (a), NH5 (b), and EV (empty vector control) (c). The final concentration of total RNA in water was 200 ng/μL. Over 100 germinated or ungerminated spores were counted and over 50 hyphae were measured from the germinated spores to quantify the effects of dsRNA on spore germination (C) and hyphal growth (D). Scale bar represents 20 μm (**A**) and 40 μm (**B**). The bar represents mean values ± standard error of the mean of one representative experiment. One-way ANOVA followed by Tukey's multiple comparison test was done. Bars with different letters are significantly different determined at P≤0.05.

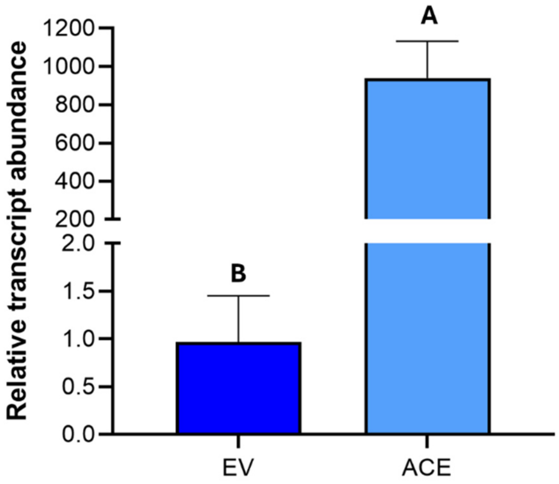

Figure S2: Relative expression of ACE amplified from another qPCR primer set. One-way ANOVA followed by Tukey's multiple comparison test was done. Bars with different letters are significantly different determined at P≤0.05.

Table S1: Primers used for amplification, cloning of the target gene fragments from *P. pachyrhizi,* for quantification of fungal biomass, and for quantification of target gene expression.

Genes	Primer name	Primer sequence (5'-3')
S5	SIGS5F_SacI	GCGgagctcACAAGAGTGACGATGAAAGAGAC
S5	SIGS5R_XhoI	CCGctcgagTGCACTACCTTGTGCGGTAA
S9	SIGS9F_SacI	GCGgagctcGAGCCGCCTGGAAAATGAAG
S9	SIGS9R_XhoI	CCGctcgagACGAAAGGGCTGCAATGAAAC
S10	SIGS10F_SacI	GCGgagctcCTAGTCTCTTATGGCGGCGG
S10	SIGS10R_XhoI	CCGctcgagGCGTTTGCTTGTCGTTCAGT
S12	SIGS12F_SacI	GCGgagctcTGGAAGGGATCAATGACGGG
S12	SIGS12R_XhoI	CCGctcgagACCAAGTGTCCAGCTGCTAA
ACE	ACEF_SacI	GCGgagctcGTTTCCACTGATGATGGCATTC
ACE	ACER_XhoI	CCGctcgagCTAGCAAGCCAAACCATAAGC
NH5	NH5F_SacI	GCGgagctcAGTATGTCTAACCCGGGCAA
NH5	NH5R_XhoI	CCGctcgagGATCACGAAAGGGCTGCAAT
NH8	NH8F_SacI	GCGgagctcAGTTCGATGGCTGGCAGTAT
NH8	NH8R_XhoI	CCGctcgagATATGCCCATCTACACCGGG
CYTB1	CYTBF1_SacI	GCGgagctcGGATGTAGAGTACGGGTGACTA
CYTB1	CYTBR1_XhoI	CCGctcgagTGATGCCGGTGTCTGTATTG
CYTB2	CYTBF2_SacI	GCGgagctcGTTCGTACAAGTCACCAAGGA
CYTB2	CYTBR2_XhoI	CCGctcgagCCTGTTACACCCAGTGGATTAC
CYP3	CYPF3_SacI	GCGgagctcCAAGGACGGCAGAGCTTTAT
CYP3	CYPR3_XhoI	CCGctcgagCCTCCTGTTCCTGCTTATCTTT
CYP4	CYPF4_SacI	GCGgagctcCAGAGGTCAATGCCGAAGAA

CYP4	CYPR4_XhoI	CCGctcgagTAGTGTGTTGACCAGCCATAAG
S12	S12_F_qPCR	CACTCAACTCAGGGAAGCAA
S12	S12_R_qPCR	GGAGTAACCATATGCCCATCTAC
CYTB1	CYTB1_F_qPCR	GATCGATTCCGAACAAGCTACT
CYTB1	CYTB1_R_qPCR	TTGACTCCCTCTTACCCTACTC
ACE	ACE_F_qPCR	CCAGGTAGCAACCGCTTTAT
ACE	ACE_R_qPCR	TTCCTGAGCCAACACACATAG
ACE	ATC_F_qPCR	GAGGAGCTGCAAATGGGTGA
ACE	ATC_R_qPCR	GAATGGGGATGGCAGCACTA
α-tubulin	TubF	CCAAGGCTTCTTCGTGTTTCA
α-tubulin	TubR	CAA GAG AAG AGC GCC AAA CC
α-tubulin	Probe	/56-FAM/TCGTTTGGA/ZEN/GGCGGACTGGTTCA/3IABkFQ/
Ubiquitin	UbqF	GACCAGCAGAGGCTGATTT
Ubiquitin	UbqR	GGACAAGGTGAAGGGTTGAT
Ubiquitin	Probe	/5SUN/TGGATGTTG/ZEN/TAGTCAGCGAGGGTC/3IABkFQ/

*restriction sites in lower case letters
